# Intestinal inflammation disrupts energy metabolism in layer pullets: insights into energy partitioning and intestinal metabolomic profiling

**DOI:** 10.1186/s40104-025-01204-x

**Published:** 2025-05-26

**Authors:** Qiuyu Jiang, Bingjian Huang, Simiao Chen, Lihua Zhao, Zhibin Ban, Bingkun Zhang

**Affiliations:** 1 State Key Laboratory of Animal Nutrition and Feeding, College of Animal Science and Technology, China Agricultural University, Beijing, 100193 China; 2 Institute of Animal Nutrition and Feed Sciences, Jilin Academy of Agricultural Sciences, Gongzhuling, Jilin, 136100 China

**Keywords:** Adenosine triphosphate, Dextran sulfate sodium, Heat production, Inflammation, Metabolomic

## Abstract

**Background:**

Intestinal inflammation is an energy-consuming process that may alter energy supply and demand in poultry. During inflammation, the intestinal energy metabolic profile and the patterns of energy partitioning remain unclear. This study investigated the effects of intestinal inflammation on energy intake, heat production (HP), retained energy (RE) and intestinal energy metabolites in layer pullets.

**Methods:**

After 7 d dietary adaption, 32 “Jing Tint 6” layer pullets with average body weight (1,123.50 ± 8.55 g) were selected from 96 birds, and randomly assigned to two groups (CON: Control group, INFL: Inflammation group) with 8 replicates per group. Indirect calorimetry analysis was conducted over 7 d to determine HP and fasting HP (FHP). During this period, pullets in INFL group received 4 mL/d of 0.6 g/mL dextran sulfate sodium (DSS) via oral gavage to induce intestinal inflammation. After the calorimetry, intestinal tissues were collected post-euthanasia from one bird per replicate for morphological and mucosal metabolomic analysis.

**Results:**

Birds exhibited significantly lower apparent metabolizable energy (AME) intake (*P* < 0.001) during intestinal inflammation, accompanied by compromised RE and RE as fat (*P* < 0.001), suggesting that birds consumed body energy to sustain energy demands. Targeted metabolomic studies identified 11 energy metabolites differentially expressed in ileal mucosa between CON and INFL groups. Specifically, DSS induction significantly increased (*P* < 0.05) adenosine triphosphate (ATP) level and reduced (*P* < 0.001) nicotinamide adenine dinucleotide (NAD^+^) level in ileal mucosa of pullets. In parallel, metabolic adaptations such as enhanced glycolytic intermediates, reduced amino acids, α-ketoglutarate (α-KG) accumulation and suppressed expression of genes encoding enzymes involved in tricarboxylic acid (TCA) cycle were observed in the inflamed ileum of pullets.

**Conclusion:**

Immune stimulation by DSS induced a negative energy balance in layer pullets, characterized by reduced AME intake (−190.47 kJ/kg BW^0.75^) and compromised RE (−18.81% of AME intake). Disruption of intestinal energy profiling was observed in inflammation-challenged pullets, such as accumulation of α-KG and ATP, reduced NAD^+^ and amino acids, which could provide valuable insights for developing effective intervention strategies.

**Supplementary Information:**

The online version contains supplementary material available at 10.1186/s40104-025-01204-x.

## Background

Intestinal inflammation, caused by various factors in commercial production, significantly challenges layer pullets by hindering their growth, delaying the onset of first laying, and negatively impacting their laying performance later in life [1]. Intestinal inflammation is an energy-intensive process, because energy substrates are required to defend and repair tissues during the immune response [2]. In normal situation, gastrointestinal tract accounts for 23% to 36% of whole-body oxygen (O_2_) consumption to support rapid cell turnover and maintain mucosal function in poultry [3, 4]. Inflamed intestine inevitably compromises the available energy and affects energy expenditure in the gastrointestinal tract and other organs. However, the characteristic and implications of energy metabolism following an acute inflammatory flare of layer pullets remain unclear.

Glucose, fatty acids and amino acids are major energy sources for the intestine of poultry [5]. Oxidation of nutrients mainly occurs via tricarboxylic acid (TCA) cycle and oxidative phosphorylation pathway to produce energy. Energy status of the intestine can be reflected by adenosine triphosphate (ATP) pool, total adenine nucleotides (TAN) and adenylate energy charge (AEC). Nicotinamide adenine dinucleotide (NAD), a major coenzyme in bioenergetic processes, receives high-energy electrons from glycolytic and TCA intermediates and eventually feeds electrons into electron transport chain (ETC) to drive ATP production [6]. In general, intestinal inflammation is regarded as an energy-deficient process involving mitochondrial disturbances that decrease ETC activity and ATP concentrations [7]. In a localized hypoxic environment created by inflammation, metabolism shifts from oxidative phosphorylation towards anaerobic glycolysis to supplement energy [8]. Immune stimulation in piglets led to increased glucose utilization, serving as primary contributor to the rise in energy expenditure [9]. Moreover, the lipid metabolism was altered following dextran sulfate sodium (DSS) induction in laying hens [10]. Currently, dietary supplementation with energy substrates such as α-ketoglutarate (α-KG), aspartate and butyrate has been shown to alleviate inflammation and restore intestinal health in livestock [7, 11, 12]. However, the comprehensive effects of immune stimulation on intestinal metabolomic profiling related to energy metabolism in layer pullets remain unclear.

Heat is considered the energy released during the oxidation of carbohydrates, fats and proteins [13]. Both the conversion of nutrients to ATP and the ATP utilization result in heat production (HP) [14]. It is reasonable to expect that intestinal inflammation may alter HP and divert energy away from animal growth. HP could be noninvasively determined based on O_2_ consumption and carbon dioxide (CO_2_) production [15]. Retained energy (RE), the difference between energy intake and HP, enables a more accurate evaluation of available energy to support growth [16]. There are limited studies investigated the correlations between intestinal inflammation and energy partitioning in livestock. In piglets, immune stimulation increased HP and reduced RE, resulting in compromised daily weight gain [17]. Understanding the extent to which energy partitioning is altered in pullets experiencing intestinal immune stimulation may help develop effective feeding strategies for health challenged birds.

Identifying how energy metabolism is altered during inflammation with a DSS challenge model might help explore new therapeutic targets to support mucosal healing. This study aimed to investigate changes in intestinal energy metabolism in layer pullets following DSS induction and to identify the associations between whole-body energy balance and intestinal inflammation.

## Materials and methods

### Diets and animal management

A corn and soybean meal-based diet was formulated (apparent metabolizable energy, AME = 11.30 MJ/kg; Crude protein, CP = 16.00%) according to the feeding standard for “Jing Tint 6” layer pullets by using software VF123 for 2024 (Jinmu Times Technology Co., Ltd., Beijing, China) [18], as shown in Table 1. A total of 96 “Jing Tint 6” layer pullets at 14 weeks of age were housed in a shed with 2 birds per pen to adapt to the basal diet. After 7 d dietary adaptation, 32 birds with average body weight of 1,123.50 ± 8.55 g were divided into two groups: Control group (CON) and Inflammation group (INFL), with 8 replicates per group, and transferred to indirect calorimetry chambers, with 2 birds per chamber. Chamber doors were opened at 9:00 each morning for feeding, fecal collection and DSS administration, and daily feed intake and body weight of birds were recorded.

**Table 1:** Ingredient composition and nutrient content of the basal diet (as-fed basis)

Items	Content
Ingredients, %	
Corn	61.33
Soybean meal (Crude protein 44%)	18.76
Wheat bran	15.77
Dicalcium phosphate	1.80
Limestone	1.35
Choline chloride (50%)	0.10
Vitamin and mineral premix^1^	0.34
Salt	0.30
DL-Methionine	0.15
Lysine HCl (78%)	0.10
Total	100.00
Calculated nutrient content, %^2^	
Apparent metabolizable energy, MJ/kg	11.30
Crude protein	16.00
Lysine	0.82
SID Lysine	0.73
Methionine	0.41
SID Methionine	0.39
Calcium	1.10
Available phosphorus	0.55
Analyzed nutrient content, %	
Gross energy, MJ/kg	15.99
Crude protein	15.87
Calcium	1.06
Total phosphorus	0.69

*SID* Standard ileal digestible

^1^Mineral premix provided the following per kg of diets: Cu 8 mg, Zn 75 mg, Fe 80 mg, Mn 100 mg, Se 0.15 mg, I 0.35 mg. Vitamin premix provided the following per kg of diets: VA 9,500 IU, VD 362.5 μg, VE 30 IU, VK_3_ 2.65 mg, VB_1_ 2 mg, VB_2_ 6 mg, VB_6_ 6 mg, VB_12_ 0.025 mg, biotin 0.0325 mg, folic acid 1.25 mg, pantothenic acid 12 mg, nicotinic acid 50 mg

^2^Nutrient contents were calculated by using software VF123 for 2024 (Jinmu Times Technology Co., Ltd., Beijing, China)

### Immune challenge

DSS with molecular weight of 36–50 kDa (CD4421; Coolabor Technology Co., Ltd., Beijing, China) was given daily at 4 mL of 0.6 g/mL via oral gavage to INFL group for 8 d to induce intestinal inflammation. It is important to mention that in the preliminary experiment, a lower DSS dose of 0.9 g/bird/d as documented in other studies [19, 20], was proven insufficient to induce inflammation in pullets, possibly due to differences in DSS manufacturers, animal breeds, and growth stages. Moreover, pullets in the CON group received a same volume of 0.9% NaCl.

### Indirect calorimetry

The calorimetry experiment was conducted for 7 d, including 2 d for environmental adaptation, 4 d for feeding measurements and the final day for fasting measurements. Open-circuit respiration chambers was previously described by Jiang et al. [21]. Chambers were air-conditioned to maintain a constant temperature of 22 to 24 °C and equipped with automatic lighting that operates on a 9-h daily cycle. A zirconium oxide sensor (Model 65-4-20; Advanced Micro Instruments Inc., Huntington Beach, CA, USA) was utilized for O_2_ detection, while a non-dispersive infrared sensor (AGM 10; Sensors Europe GmbH, Erkrath, Germany) was employed for CO_2_ detection. Real time O_2_ and CO_2_ were used for HP and FHP analysis, following the equation originally proposed by Brouwer [22] as follows:

$$\mathrm{HP}(\mathrm{kJ}/\mathrm d)=16.18\times\mathrm O_2(\mathrm L/\mathrm d)+5.02\times\mathrm{CO}_2(\mathrm L/\mathrm d)$$

Moreover, respiratory quotient (RQ) represents the ratio of CO_2_ produced to O_2_ consumed. Heat increment (HI) can be calculated by subtracting FHP from HP.

### Fecal scoring, collection and chemical analysis

On d 4, 5, 6 and 7 post-infection, droppings were evaluated for consistency using a scale from 0 to 4 according to the previous studies [23, 24], with minor modifications to include an additional score for bloody stools. After scoring, excreta were collected for 4 d, thoroughly homogenized, oven-dried at 65 °C for 72 h, and then ground through a 1-mm screen. All chemical analyses of feed and excreta were conducted in duplicate on a dry matter (DM) basis according to AOAC (2006) [25]. Nitrogen content was determined (method 984.13) with a Kjeltec Distillation Unit (KT 200 101; FOSS Analytical, Hillerød, Denmark), and derived values were then multiplied by 6.25 to obtain crude protein (CP). Calcium was measured by titration with 0.03 mol/L KMnO_4_, and total phosphorus was determined colorimetrically using a molybdovanadate reagent (method 965.17). Samples were analyzed for gross energy (GE) content using a bomb calorimeter (IKA-C3000, Staufen, Germany). The AME intake is calculated by subtracting GE excretion from GE intake, while retained protein is the difference between CP intake and CP excretion. Energy retained as protein (RE as protein) is calculated by multiplying retained protein by 23.86 kJ/g, which represents the energy equivalent of 1-g protein [26]. Moreover, energy retained as fat (RE as fat) is calculated by subtracting RE as protein from RE.

### Sampling and intestinal mass measurement

One bird per chamber was randomly selected on d 7 of the experiment, and blood was collected from brachial vein and allowed to clot at room temperature for 4 h. Followed by centrifugation at 3,000 × *g* for 15 min, the obtained serum was stored at −20 °C for further analysis. Birds were euthanized at 9:00 on d 8 of the experiment. The entire intestine was carefully collected, and the intestinal weight and lengths of each intestinal segment were measured. The intestinal segments were then dissected and spread on a white background for intestinal integrity analysis. The jejunum, extending from the end of the duodenum to Meckel’s diverticulum, and the ileum, starting from Meckel’s diverticulum to ileocecal junction were excised. Approximately 1-cm length midsections of the jejunum and ileum were carefully collected and fixed in 4% paraformaldehyde for histological analysis. Additional 2-cm segments from the midsection of the folded jejunum and ileum were collected, cut into small pieces, and the ileal mucosa was scraped from the remaining tissues using sterilized microscope slides. The rinsed mid-jejunum, mid-ileum and mucosa were immediately immersed in liquid nitrogen and stored at −80 °C for further analysis.

### Intestinal morphology and histological score

The intestinal integrity index, a quantitative tool developed by a global company (Elanco Animal health, Greenfield, USA) was used to assess intestinal health for poultry [27]. Intestinal health conditions were simplified from 23 to 7 key types: intestinal tone, excessive intestinal fluid, thin intestines, thick intestines, excessive mucus, hyperemia, and intestinal hemorrhage. Lesion scores for each condition vary from 0 to 3 and each condition is weighted equally, contributing to a total score of 100.

The hematoxylin–eosin stained slides were scanned using a microscope (TE2000; Nikon, Tokyo, Japan) and visualized with ImageJ software (National Institutes of Health, Bethesda, MD, USA) to assess villus height, crypt depth and the villus height to crypt depth ratio (VCR). Blind scoring of jejunal and ileal sections was performed according to the published system [11]. The overall histological score was the sum of four variables: severity of inflammation (Score: 0–3), extent of inflammation (Score: 0–3), percent involvement (Score: 0–4) and crypt damage (Score: 0–4), with a maximum score of 14.

### Serum parameters and nicotinamide adenine dinucleotide content

The D-lactate and diamine oxidase levels in serum were measured by using commercial kits (Elabscience, Wuhan, China) with a SpectraMax i3x multi-mode Microplate Reader (Molecular Devices; San Jose, CA, USA) at wavelengths of 530 nm and 460 nm, respectively.

NAD⁺ and NADH contents in ileal mucosa were determined at a wavelength of 450 nm using a commercial kit (E-BC-K804-M; Elabscience, Wuhan, China). Particularly, a 10 kDa ultrafiltration tube (FUF051; Beyotime Biotechnology, Beijing, China) was used during centrifugation to remove the degradation enzymes. Protein content of ileal mucosa was determined using a Pierce BCA kit (23227; Thermo Fisher Scientific, Waltham, MA, USA).

### Real-time quantitative PCR

Approximately 0.3 g of ileal tissue was homogenized in 1 mL of TRIzol reagent (Genstar, Beijing, China) to extract total RNA, and its concentration and purity were assessed using a NanoDrop 2000 spectrophotometer (Thermo Fisher Scientific, Waltham, MA, USA). The cDNA was synthesized from 1,000 ng of total RNA using a kit with gDNA eraser (RR092 A; Takara Bio Inc., Shiga, Japan). The SYBR Green qPCR master mix (Q311-02; Nuoweizan, Nanjing, China) was used for real-time qPCR analysis, following the recommended amplification conditions. Table 2 presents the primer sequences for gene amplification. Target gene expression, normalized to the housekeeping gene β-actin (*ACTB*), was calculated and analyzed using the 2^−ΔΔCt^ method.

**Table 2:** Primer sequences used in the real-time qPCR

Items	Primer sequence (5′→3′)	GenBank accession No.
*TJP1*	F: CTTCAGGTGTTTCTCTTCCTCCTC	XM_046925210.1
	R: CTGTGGTTTCATGGCTGGATC	
*CLDN1*	F: GGTATGGCAACAGAGTGGCT	NM_001013611.2
	R: CAGCCAATGAAGAGGGCTGA	
*OCLN*	F: ACGGCAGCACCTACCTCAA	NM_205128.1
	R: GGGCGAAGAAGCAGATGAG	
*LGR5*	F: TCAATACCTGAGCGTGCGTT	XM_040662215.2
	R: TGTGAGTGTCAAACTCTCCAGAC	
*MUC2*	F: ACCACCACAACACCCTTCAG	XM_040701656.2
	R: TACTGGTTGCGAAGGTGGAG	
*HK*	F: AGCTCCTGGCCTACTACTTCA	NM_204101.2
	R: ACCGAGATCCAGAGCGATGA	
*PFK*	F: TGGCCACACCATCTATGCAG	NM_001396039.1
	R: TTTCCGCACATTCTCCACGA	
*PGK2*	F: GGGTCGTCATGAGGGTTGAC	NM_204985.4
	R: GGCCCAGGTGACTCATCAAA	
*PK*	F: GGAGTGGGCGCTTATGGTAA	NM_205469.2
	R: TTGTAGGAACCGTGGAGCAC	
*CS*	F: GGAACGGGCGTTGTTTCGG	XM_046905018.1
	R: TGCGGGCGCTGATTCATTA	
*IDH3A*	F: GACCAGAAGTTTTAGTAGTGCTGT	NM_001005808.3
	R: AGGTTTTACGCAGCAGCAGA	
*α-KGDH*	F: TTCAAGCACAGCCCAACGTA	NM_001031382.2
	R: GCCCGTAAAATCCGACGTTT	
*SUCLA2*	F: TACGATTGCAAGGTACGCGA	NM_001006271.2
	R: CCAAGTCGTCACAGGCAAGA	
*SDHB*	F: GGTCCAGGGGATCTGTCG	NM_001080875.3
	R: GTTCATTGCACAGGAGCCAC	
*ATP5A*	F: GGCAATGAAACAGGTGGCAG	XM_046934479.1
	R: GGGCTCCAGCTTGTCTAAGTGA	
*ATP5B*	F: AAGGCTCCATCACTTCGGTG	NM_001031391.3
	R: TGTTGGGGTCCATGATTCGG	
*ACTB*	F: CAACACAGTGCTGTCTGGTGGTAC	NM_205518.2
	R: CTCCTGCTTGCTGATCCACATCTG	

*F* Forward, *R* Reverse

### Metabolomic profiling

Targeted metabolomic profile was evaluated using ultra-performance liquid chromatography (UPLC)-tandem mass spectrometry (MS/MS), as previously described by Jiang et al. [28]. Briefly, 50 mg of ileal mucosa was homogenized in 100 μL of ddH_2_O and mixed with 500 μL of pre-cooled methanol/ddH_2_O (7:3, vol/vol). Following centrifugation at 14,000 × *g* for 10 min, the supernatant was incubated at −20 °C for 30 min and centrifuged again. The obtained supernatant was used for UPLC-MS/MS analysis. The UPLC (Waters ACQUITY H-ClassD, Milford, USA) with a 2.1 mm × 100 mm ACQUITY UPLC BEH Amide column (internal diameter 1.7 μm; Waters, Milford, USA) heated to 45 °C was used for analysis. The mobile phases consisted of two solutions: (A) H_2_O (10 mmol/L ammonium acetate and 0.3% ammonia) and (B) acetonitrile/H_2_O (9:1, vol/vol). MS/MS analysis (QTRAP 6500 +, SCIEX) was performed with an electrospray ionization temperature of 550 °C. The ATP, adenosine diphosphate (ADP), and adenosine monophosphate (AMP) concentrations were used to calculate the TAN and AEC, as described by Newman et al. [29]:

$$TAN = AMP + ADP + ATP$$

$$AEC = (0.5 \times ADP + ATP)/(AMP + ADP + ATP)$$

### Data processing and statistical analyses

The chamber (two birds) was treated as the experimental unit. All data were analyzed using independent sample *t*-test process in SPSS software 24.0 (SPSS Inc., Chicago, USA). A homogeneity of variances test was performed, and based on the results, either the *t*-test assuming equal variances or Welch’s *t*-test was applied. Results are considered statistically significant at *P* < 0.05 and as trends at 0.05 ≤ *P* < 0.10. Results are presented as mean ± SEM (standard error of the mean), and metabolite concentrations are shown as ranges from the minimum to the maximum. All data were visualized using GraphPad Prism (8.0.2; San Diego, CA, USA). R software (R Core Team, Vienna, Austria) was used for metabolomic data processing, with thresholds set at fold change (FC) > 2 or < 0.5 and variable importance in projection (VIP) > 1.

## Results

### Evaluation of intestinal inflammation

**Fig. 1:**
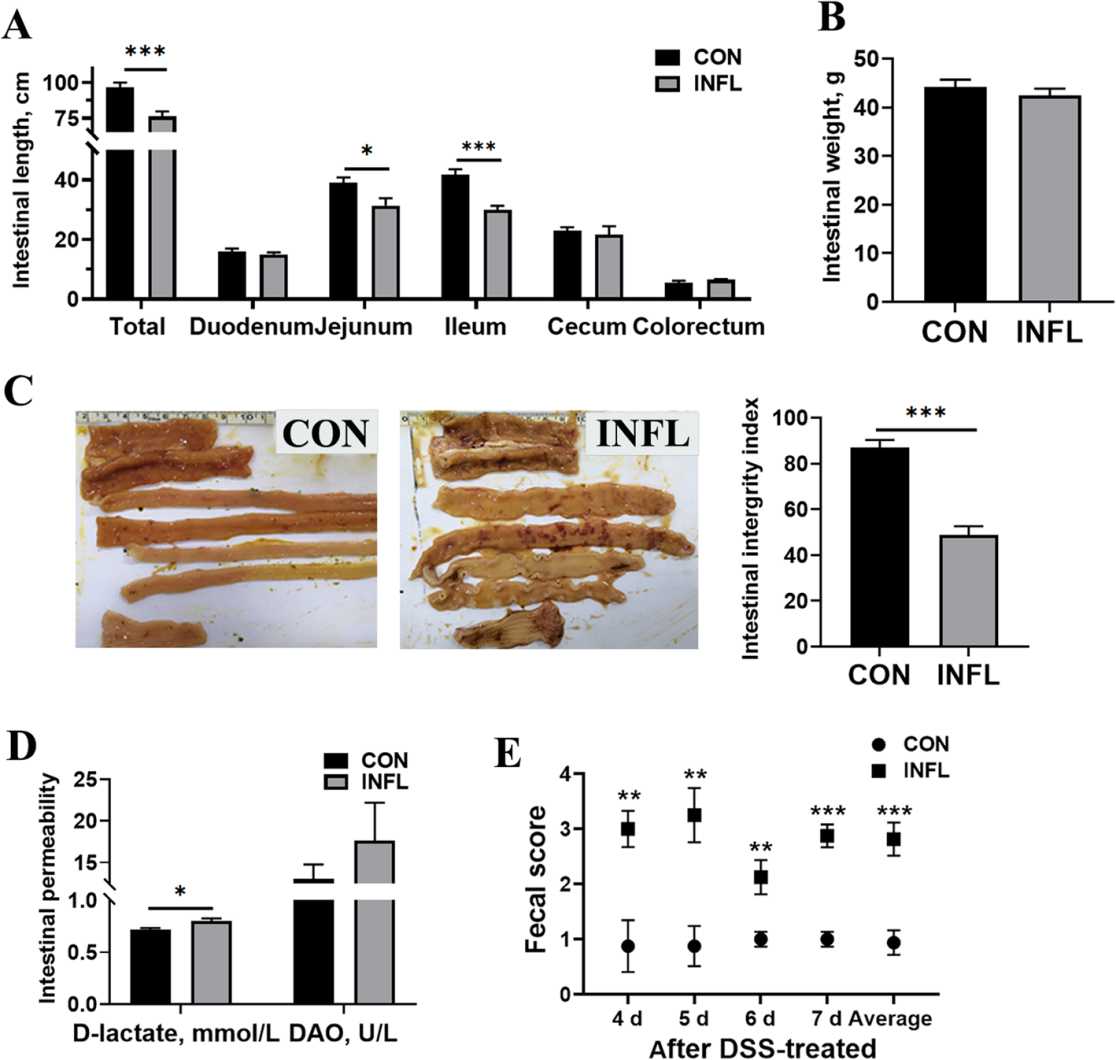
Evaluation of the intestinal inflammation model induced by DSS in layer pullets. **A** The lengths of duodenum, jejunum, ileum, cecum, colorectum and total intestine. **B** Intestinal weight. **C** Intestinal integrity index of the intestine, with a maximum score of 100. **D** Intestinal permeability-related indices including D-lactate and diamine oxidase in serum. **E** Fecal score of layer pullets on d 4, 5, 6 and 7 post-DSS induction, as well as the average score. CON and INFL represent control group and inflammation group, respectively. Results are presented as mean ± SEM (*n* = 8). ^*^P < 0.05; ^**^P < 0.01; ^***^P < 0.001

DSS administration for 8 d induced inflammatory symptoms such as shortened intestinal length and impaired integrity, as presented in Fig. 1A–C. Significant reductions in the lengths of the jejunum, ileum and total intestine were observed (*P* < 0.05) in layer pullets in the INFL group. The integrity index was significantly decreased (*P* < 0.001) in the INFL group, characterized by weakened intestinal tone, thickened intestines and intestinal hemorrhage, as indicated in Fig. 1C. In parallel, these birds exhibited significantly higher D-lactate levels in serum (*P* < 0.05), suggesting that DSS induced intestinal leakage to some extent (Fig. 1D). The fecal score of pullets was severely increased at d 4, 5, 6 and 7 following DSS induction (*P* < 0.05) compared to untreated birds (Fig. 1E). In detail, loose feces with reduced uric acid, accompanied by bloody stools were observed in pullets in the INFL group.

**Fig. 2:**
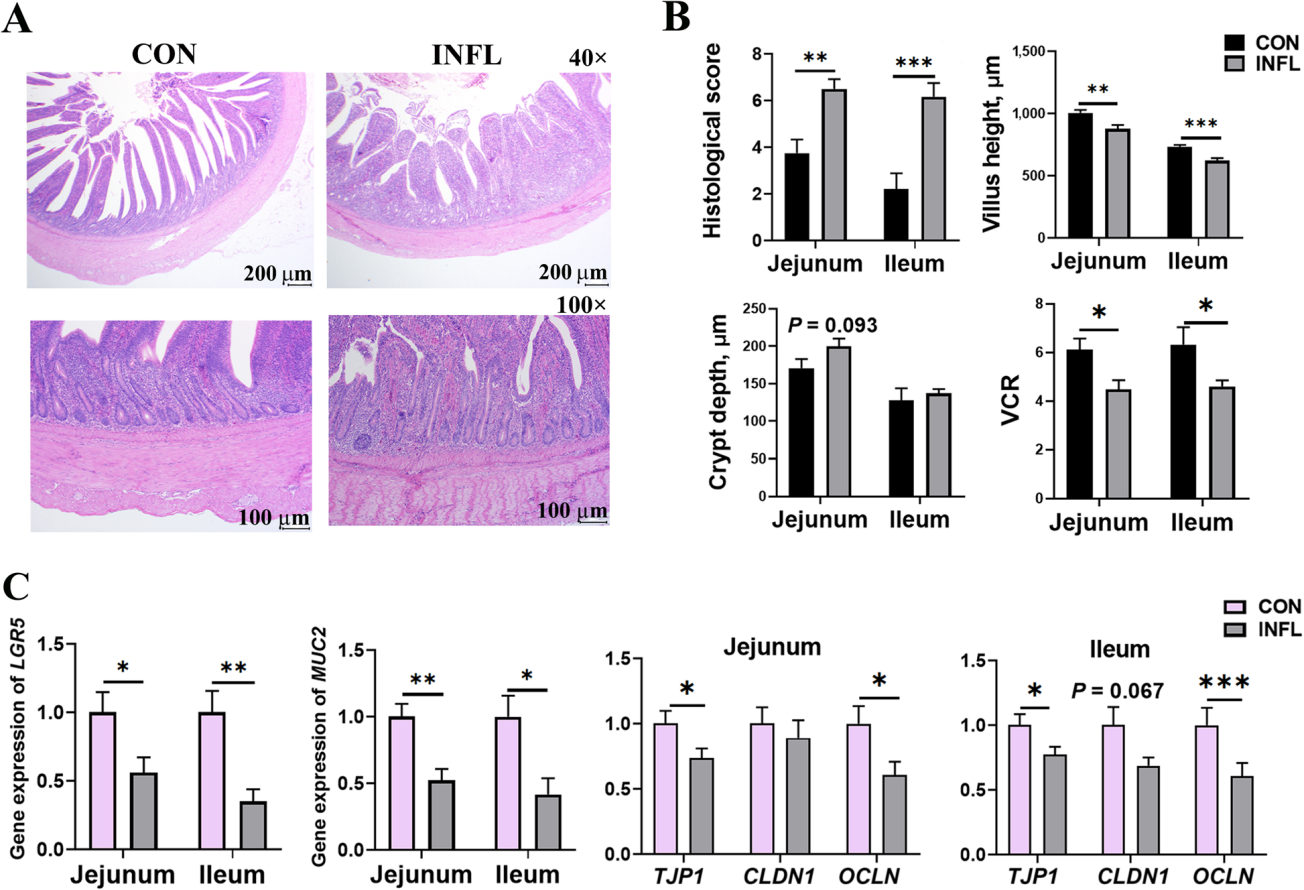
Intestinal histological analysis and expression of genes associated with barrier functions in jejunum and ileum of layer pullets. **A** Hematoxylin–eosin stained intestinal sections of ileum. Scale bars represent 200 µm for 40 × magnification and 100 µm for 100 × magnification, respectively. The 100 × image of the inflamed ileum reveals histological lesions, including inflammatory cell infiltration and hemorrhage in the lamina propria, disrupted crypt architecture, and transmural inflammation. **B** Histological score (with a maximum score of 14), villus height, crypt depth and villus height to crypt depth ratio of jejunum and ileum. **C** Relative gene expression of *LGR5*, *MUC2*, *TJP1*, *CLDN1* and *OCLN* in jejunum and ileum. CON and INFL represent control group and inflammation group, respectively. Results are presented as mean ± SEM (*n* = 8). ^*^P < 0.05; ^**^P < 0.01; ^***^P < 0.001

Figure 2A and B illustrate impaired intestinal morphology and increased histological scores in inflamed jejunum and ileum of pullets. DSS induced reductions (*P* < 0.05) in villus height and VCR, but no significant differences were observed in crypt depth between the two groups for the jejunum (*P* = 0.093) and ileum (*P* > 0.05). Specifically, the histological characteristics of the inflamed intestine showed disrupted crypt architecture, infiltration of inflammatory cells, hemorrhage in lamina propria and even transmural involvement (Fig. 2A). As expected, these histological alterations were associated with marked downregulation of genes (*P* < 0.05) encoding the stem cell marker Leucine-rich repeat-containing G-protein coupled receptor 5 (*LGR5*) and goblet cell marker Mucin 2 (*MUC2*) in jejunum and ileum (Fig. 2C). Gene expression of tight junction proteins, which connect enterocytes and regulate intestinal permeability is shown in Fig. 2C. The tight junction protein 1 (*TJP1*) and occludin (*OCLN*) expression were significantly downregulated (*P* < 0.05) in the inflamed intestine, while claudin 1 (*CLDN1*) showed a trend toward downregulation in the ileum of pullets (*P* = 0.067).

### Whole-body energy homeostasis

#### Respiratory data

Indirect calorimetry was performed to assess the impact of intestinal inflammation on energy balance in body. As shown in Table 3, body weight, O_2_ consumption and CO_2_ production of layer pullets were not affected by enteritis induction in either fed or fasted state (*P* > 0.05). Birds in the INFL group exhibited a trend to reduce feed intake after DSS induction (*P* = 0.068). Interestingly, RQ was significantly higher in DSS-treated birds (*P* < 0.05) compared to untreated birds, but only in the fasted state.

**Table 3:** Body weight, oxygen consumption, carbon dioxide production and respiratory quotient in layer pullets during intestinal inflammation

Items	CON	INFL	*P*-value
Fed state			
Body weight, g^1^	1,134.13 ± 25.74	1,106.13 ± 26.77	0.463
Feed intake, g	57.71 ± 5.44	46.13 ± 2.67	0.068
Oxygen consumption, L/d	27.02 ± 0.89	28.08 ± 1.60	0.572
Carbon dioxide production, L/d	26.21 ± 1.21	27.62 ± 1.56	0.485
Respiratory quotient^2^	0.97 ± 0.02	0.98 ± 0.01	0.448
Fasted state			
Body weight, g^1^	1,106.38 ± 23.44	1,079.75 ± 23.13	0.432
Oxygen consumption, L/d	21.22 ± 0.55	21.90 ± 1.38	0.658
Carbon dioxide production, L/d	15.75 ± 0.52	17.44 ± 1.03	0.167
Respiratory quotient^2^	0.74 ± 0.01^b^	0.80 ± 0.02^a^	0.035

CON and INFL represent control group and inflammation group, respectively

Results are presented as mean ± SEM (*n* = 8)

^1^Body weight is measured at the end of feeding and fasting experiments

^2^Respiratory quotient is defined as the ratio of carbon dioxide production to oxygen consumption

^a,b^Means in the same row with different superscripts are significantly different at *P* < 0.05

#### AME intake, heat production and retained energy

As shown in Table 4, DSS-treated birds exhibited significantly lower AME intake (*P* < 0.001) compared with those in the CON group (460.98 vs. 651.45 kJ/kg BW^0.75^/d), whereas HP, FHP and HI remained unchanged (*P* > 0.05). As a result, RE, RE as protein and RE as fat significantly decreased (*P* < 0.001) in DSS-treated birds, with RE and RE as fat dropping below zero (−86.72 and −144.64 kJ/kg BW^0.75^/d, respectively), indicating a negative energy balance in the body.

**Table 4:** Energy intake, heat production and energy retention in layer pullets during intestinal inflammation

Items, kJ/kg BW^0.75^/d	CON	INFL	*P*-value
AME intake	651.45 ± 12.48^a^	460.98 ± 28.19^b^	< 0.001
Heat production	518.40 ± 12.95	547.70 ± 31.69	0.414
Fasting heat production	391.74 ± 7.32	417.69 ± 25.67	0.359
Heat increment	126.66 ± 8.32	130.01 ± 7.44	0.768
Retained energy	133.05 ± 4.53^a^	−86.72 ± 26.70^b^	< 0.001
Energy retained as protein	110.48 ± 2.77^a^	57.92 ± 7.27^b^	< 0.001
Energy retained as fat	22.57 ± 5.52^a^	−144.64 ± 23.77^b^	< 0.001

*AME* Apparent metabolizable energy. CON and INFL represent control group and inflammation group, respectively

Results are presented as mean ± SEM (*n* = 8)

^a,b^Means in the same row with different superscripts are significantly different at *P* < 0.05

#### The partition of AME intake and HP

**Fig. 3:**
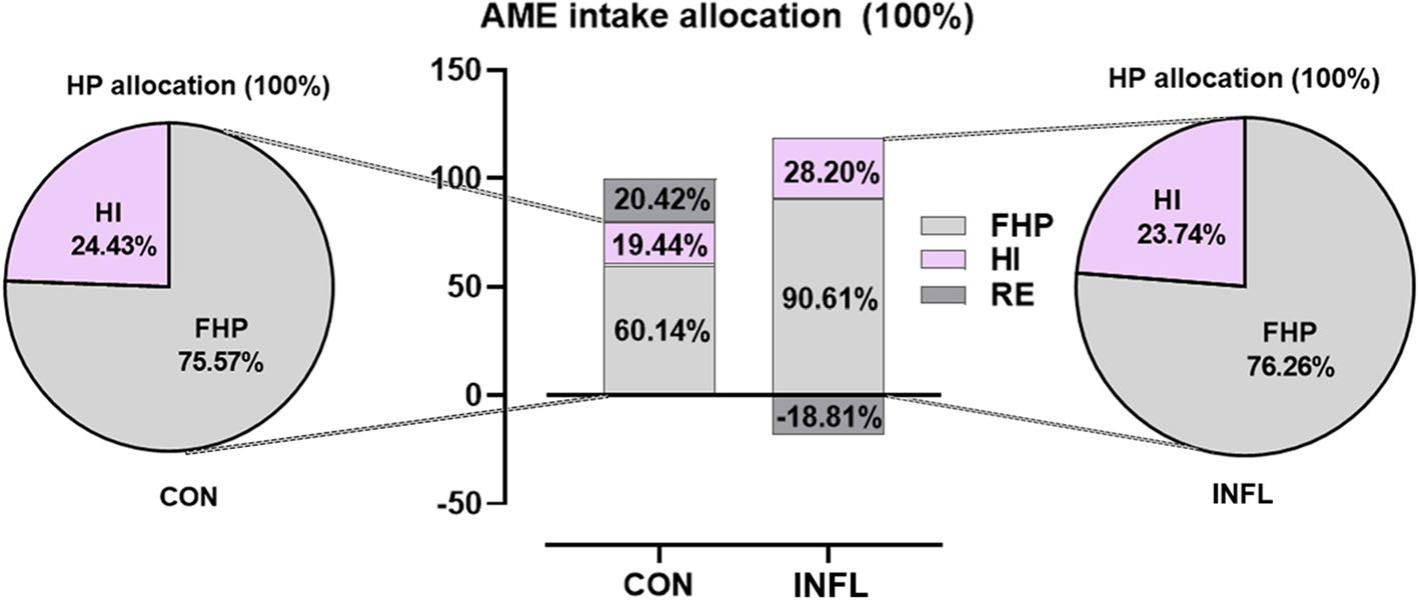
Combined pie and bar chart displaying the allocation of energy intake and heat production in layer pullets challenged with or without DSS. The bar chart displays the allocation of AME intake among FHP, HI and RE, while pie chart further illustrates the proportion of HP divided between HI and FHP. CON and INFL represent control group and inflammation group, respectively

Figure 3 illustrates the allocation of AME intake to FHP, HI and RE in a bar chart, along with the distribution of HP between HI and FHP in a pie chart. In the CON group, 60.14% of AME intake was allocated to FHP, while 19.44% and 20.42% were allocated to HI and RE, respectively. During intestinal inflammation, FHP increased to 90.61% of AME intake and HI slightly rose to 28.20%, resulting in a negative energy balance with RE accounting for −18.81% of AME intake. In the CON group, HI and FHP accounted for 24.43% and 75.57% of total HP, respectively. Similarly, these values were observed at 23.74% and 76.26% following enteritis induction.

### Energy metabolism in ileum

#### Differential metabolites and associated pathways

**Fig. 4:**
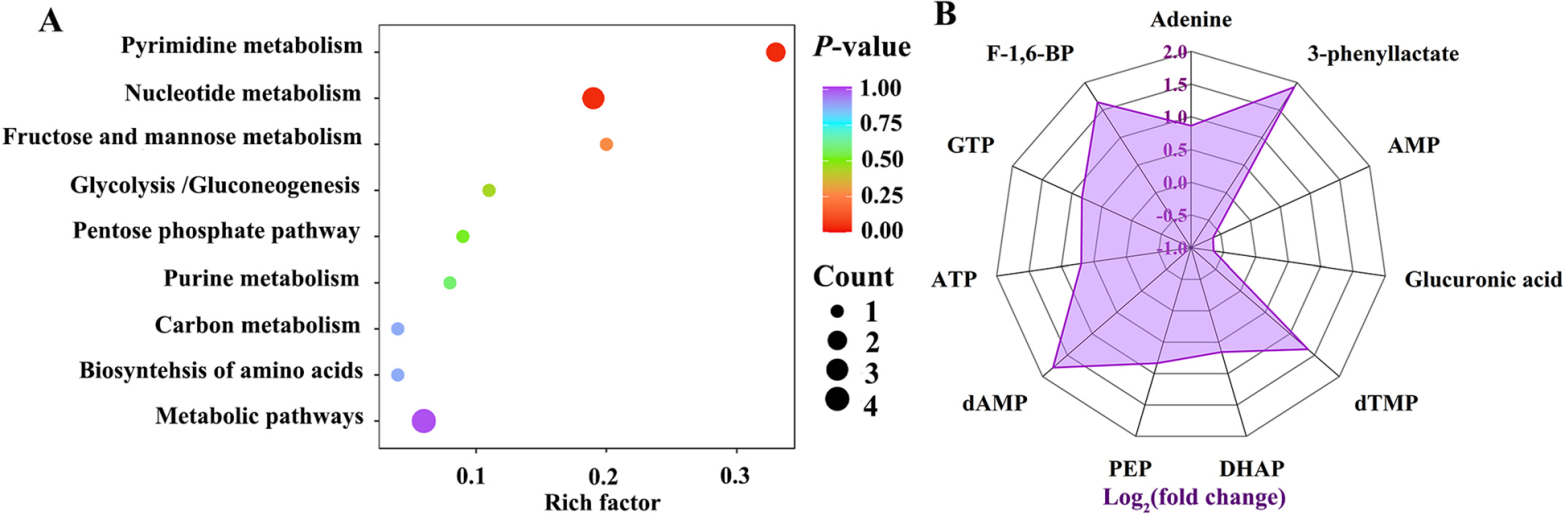
Differential energy metabolites and metabolic pathways in the ileal mucosa of layer pullets after enteritis induction. **A** KEGG enrichment analysis of energy metabolites between untreated and DSS-treated birds based on specific criteria: FC > 2 or < 0.5; VIP > 1. Count represents the number of differential metabolites in a given metabolic pathway. Rich factor is the ratio of differential metabolite numbers to total metabolite numbers annotated in that pathway. **B** Radar chart displaying the top 11 differential metabolites ranked by fold change values. Log_2_(FC) > 0 indicates an increase of metabolite levels in the INFL group, while Log_2_(FC) < 0 indicates a reduction

KEGG enrichment analysis of energy metabolism revealed that eight pathways were pronouncedly enriched in inflamed ileum, including pyrimidine, purine and nucleotide metabolism, carbon metabolism, fructose and mannose metabolism, pentose phosphate pathway (PPP), biosynthesis of amino acids as well as glycolysis and gluconeogenesis (Fig. 4A). Radar chart displays the top 11 differential metabolites with values further transformed to log_2_ (Fig. 4B). Among differential metabolites, AMP and glucuronic acid were downregulated in inflamed ileum, as indicated by log_2_(fold change) < 0. In contrast, adenine, 3-phenyllactate, deoxythymidine monophosphate (dTMP), dihydroxyacetone phosphate (DHAP), phosphoenolpyruvate (PEP), deoxyadenosine monophosphate (dAMP), ATP, guanosine triphosphate (GTP) and fructose-1,6-bisphosphate (F-1,6-BP) were enriched in ileal mucosa following enteritis induction.

#### Adenine nucleotide pool

Adenine nucleotides including AMP, ADP and ATP were selected for further analysis (Table 5). Interestingly, AMP content and AMP/ATP ratio in ileal mucosa of pullets were significantly reduced during inflammation (*P* < 0.05). A trend toward decreased TAN content was observed in ileal mucosa following DSS challenge (*P* = 0.071). Conversely, enteritis induction significantly elevated ATP content and AEC in ileal mucosa of pullets compared to CON group (*P* < 0.05).

**Table 5:** Intestinal inflammation remodels adenine nucleotide pool in ileal mucosa of layer pullets

Items	CON	INFL	*P*-value
AMP, μg/g	30.29 ± 3.16^a^	19.57 ± 2.49^b^	0.019
ADP, μg/g	2.76 ± 0.35	3.62 ± 0.43	0.142
ATP, μg/g	2.82 ± 0.38^b^	4.56 ± 0.59^a^	0.028
AMP/ATP, g/g	11.98 ± 1.57^a^	4.90 ± 1.03^b^	0.002
ADP/ATP, g/g	1.13 ± 0.26	0.91 ± 0.17	0.491
TAN, μg/g^1^	35.86 ± 3.19	27.75 ± 2.66	0.071
AEC, g/g^2^	0.12 ± 0.02^b^	0.24 ± 0.03^a^	0.001

*AMP* Adenosine monophosphate, *ADP* Adenosine diphosphate, *ATP* Adenosine triphosphate, *TAN* Total adenine nucleotides, *AEC* Adenylate energy charge. CON and INFL represent control group and inflammation group, respectively

Results are presented as mean ± SEM (*n* = 8)

^1^TAN = AMP + ADP + ATP

^2^AEC = (0.5 × ADP + ATP)/(AMP + ADP + ATP)

^a,b^Means in the same row with different superscripts are significantly different at *P* < 0.05

#### Nicotinamide adenine dinucleotide pool

As shown in Table 6, DSS induction markedly reduced NAD⁺ level, total levels of NAD⁺ and NADH, and the NAD⁺/NADH ratio in ileal mucosa of pullets (*P* < 0.01). Moreover, NADH level in ileal mucosa of DSS-treated birds was numerically increased (*P* = 0.083) compared to that in CON group.

**Table 6:** Intestinal inflammation alters the balance of NAD^+^ and NADH in ileal mucosa of layer pullets

Items	CON	INFL	*P*-value
NAD⁺, μmol/mg pro	47.25 ± 3.48^a^	24.23 ± 2.19^b^	< 0.001
NADH, μmol/mg pro	14.00 ± 0.83	16.03 ± 0.70	0.083
NAD⁺ + NADH, μmol/mg pro	61.25 ± 3.29^a^	40.26 ± 2.01^b^	< 0.001
NAD⁺/NADH	3.50 ± 0.39^a^	1.56 ± 0.19^b^	0.001

*NAD* Nicotinamide adenine dinucleotide. CON and INFL represent control group and inflammation group, respectively

Results are presented as mean ± SEM (*n* = 8)

^a,b^Means in the same row with different superscripts are significantly different at *P* < 0.05

#### Energy metabolites and enzymes involved in glycolysis

**Fig. 5:**
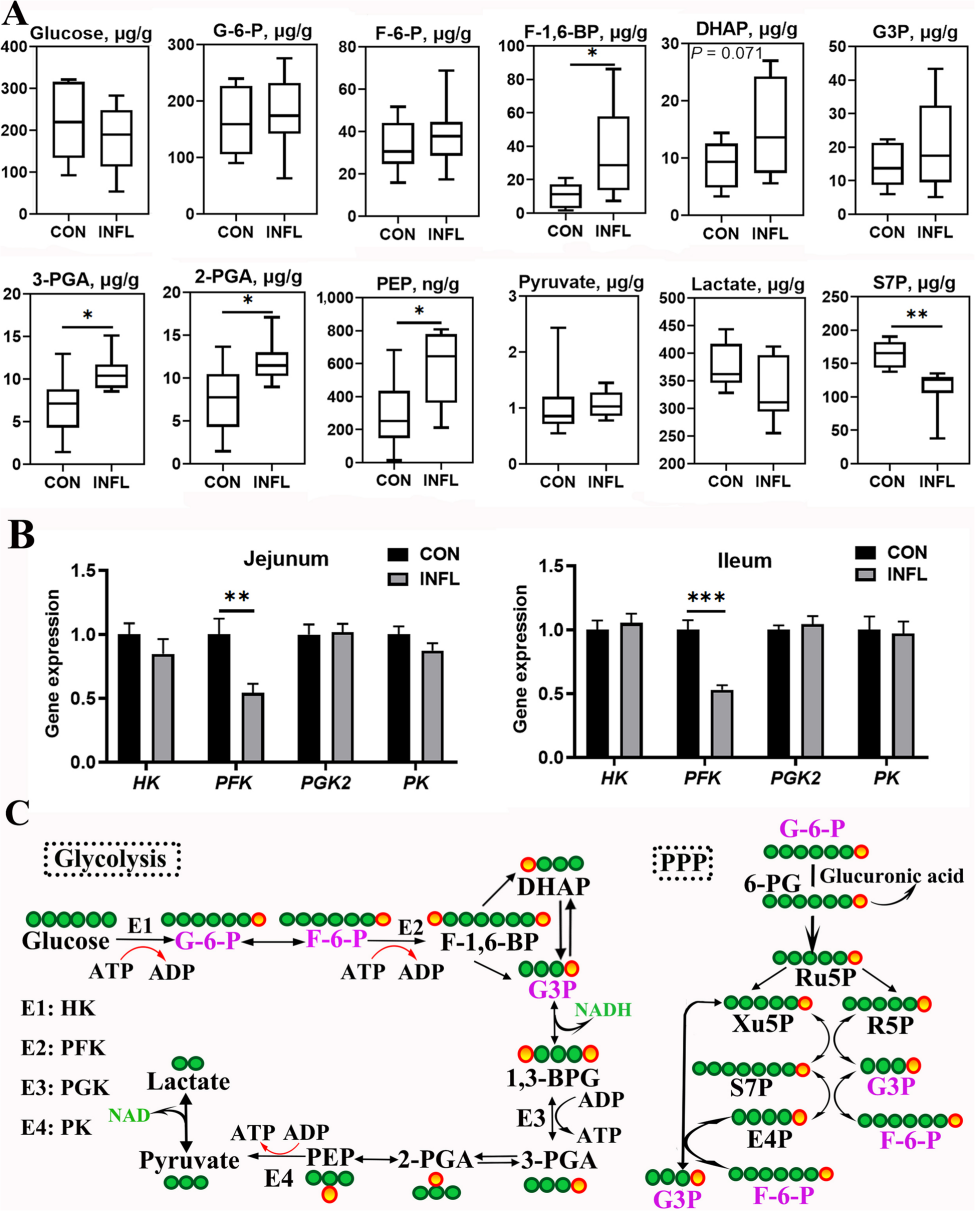
Alteration of glycolytic metabolites and enzymes in ileal mucosa of layer pullets following enteritis induction. **A** Contents of glycolytic metabolites and S7P in ileal mucosa. Results represent the range from minimum to maximum values (*n* = 8). **B** Gene expression of enzymes involved in glycolysis in jejunum and ileum. Results are presented as mean ± SEM (*n* = 8). **C** Schematic representation of the metabolites and enzymes involved in glycolysis and pentose phosphate pathway. CON and INFL represent control group and inflammation group, respectively. ^*^P < 0.05; ^**^P < 0.01; ^***^P < 0.001

As for glycolytic metabolites (Fig. 5A), DSS induction significantly increased (*P* < 0.05) contents of F-1,6-BP, 3-phosphoglycerate (3-PGA), 2-phosphoglycerate (2-PGA) and PEP in ileal mucosa of pullets, while DHAP showed numerical increase (*P* = 0.071) in ileal mucosa of birds after enteritis induction. Relevantly, sedoheptulose-7-phosphate (S7P) that involved in PPP was reduced in ileal mucosa of DSS-treated birds (*P* < 0.01). Other PPP intermediates that did not differ between the two groups were not present in this study. Figure 5B illustrates alterations in the gene levels of glycolytic enzymes in response to enteritis induction and phosphofructokinase (*PFK*) was significantly downregulated (*P* < 0.01) in jejunum and ileum of layer pullets. Figure 5C presents a schematic representation of enzymes and metabolites involved in glycolysis and PPP.

#### Energy metabolites and enzymes involved in TCA cycle

**Fig. 6:**
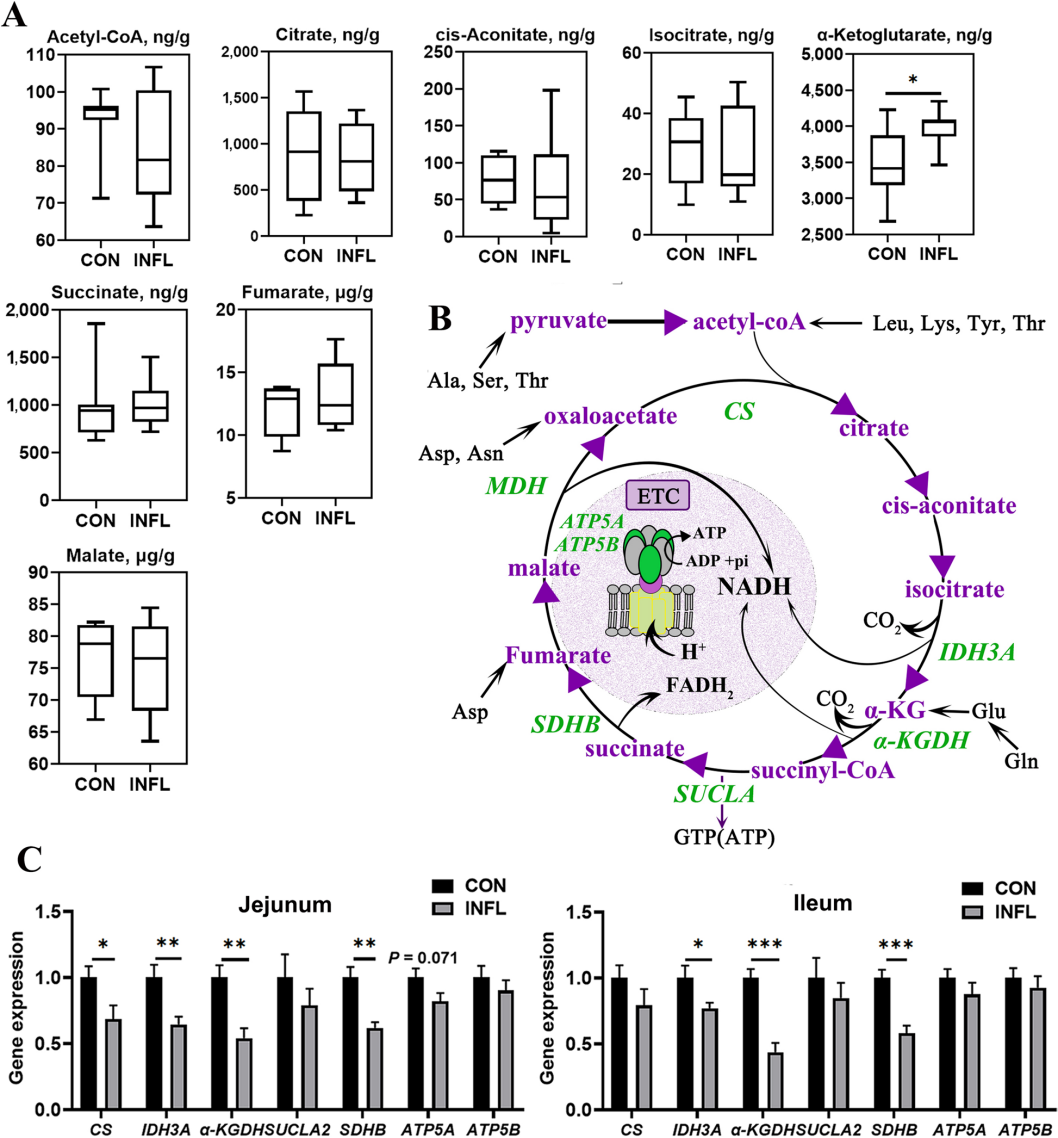
Alteration of metabolites and enzymes involved in the TCA cycle in ileal mucosa of layer pullets following enteritis induction. **A** Concentrations of metabolites involved in TCA cycle in ileal mucosa. Results represent minimum to maximum values (*n* = 8). **B** Schematic representation of the metabolites and enzymes involved in TCA cycle. **C** Gene expression of enzymes involved in TCA cycle in jejunum and ileum. Results are presented as mean ± SEM (*n* = 8). CON and INFL represent control group and inflammation group, respectively. ^*^P < 0.05; ^**^P < 0.01; ^***^P < 0.001

Regarding TCA cycle metabolites in ileal mucosa (Fig. 6A), no significant differences were observed between the two groups, except for α-KG that significantly increased following DSS induction (*P* < 0.05). Figure 6B illustrates the metabolites (in purple) and enzymes (in green) involved in TCA cycle and ETC pathway. As shown in Fig. 6C, the gene expression of isocitrate dehydrogenase (NAD^+^) 3 catalytic subunit α (*IDH3A*), α-ketoglutarate dehydrogenase complex (*α-KGDH*) and succinate dehydrogenase B (*SDHB*) was significantly downregulated (*P* < 0.05) in both the jejunum and ileum after DSS challenge. In addition, DSS-treated birds exhibited lower gene expression of citrate synthase (*CS*) (*P* < 0.05) and ATP synthase F1 subunit alpha (*ATP5A*) (*P* = 0.071) in the jejunum compared to untreated birds.

#### Abundance of amino acids in ileal mucosa

As shown in Table 7, contents of protein (*P* = 0.054), alanine (*P* = 0.036), serine (*P* = 0.010), asparagine (*P* = 0.023), threonine (*P* = 0.099), tyrosine (*P* = 0.099) and glutamine (*P* = 0.082) were severely decreased in inflamed ileum. These amino acids can be converted into intermediates of TCA cycle to generate energy, as illustrated in Fig. 6B.

**Table 7:** Intestinal inflammation reshapes protein and amino acid abundance in ileal mucosa of layer pullets

Items	CON	INFL	*P*-value
Protein, mg/g	87.23 ± 2.11	80.68 ± 2.29	0.054
Amino acids, μg/g			
Threonine	208.89 ± 13.79	179.79 ± 7.52	0.099
Leucine	304.04 ± 13.81	279.19 ± 8.18	0.160
Lysine	485.89 ± 36.05	410.60 ± 30.77	0.142
Tyrosine	175.29 ± 8.29	158.26 ± 4.51	0.099
Alanine	46.31 ± 1.95^a^	40.09 ± 1.75^b^	0.036
Serine	416.26 ± 13.62^a^	366.23 ± 8.76^b^	0.010
Glutamate	66.12 ± 1.42	67.76 ± 1.68	0.467
Aspartate	639.43 ± 33.85	595.40 ± 15.37	0.264
Asparagine	92.27 ± 4.26^a^	76.30 ± 4.46^b^	0.023
Glutamine	918.30 ± 21.97	855.98 ± 24.88	0.082
Arginine	352.79 ± 18.33	311.68 ± 18.75	0.142

CON and INFL represent control group and inflammation group, respectively

Results are presented as mean ± SEM (*n* = 8)

^a,b^Means in the same row with different superscripts are significantly different at *P* < 0.05

### Pearson correlation analysis

**Fig. 7:**
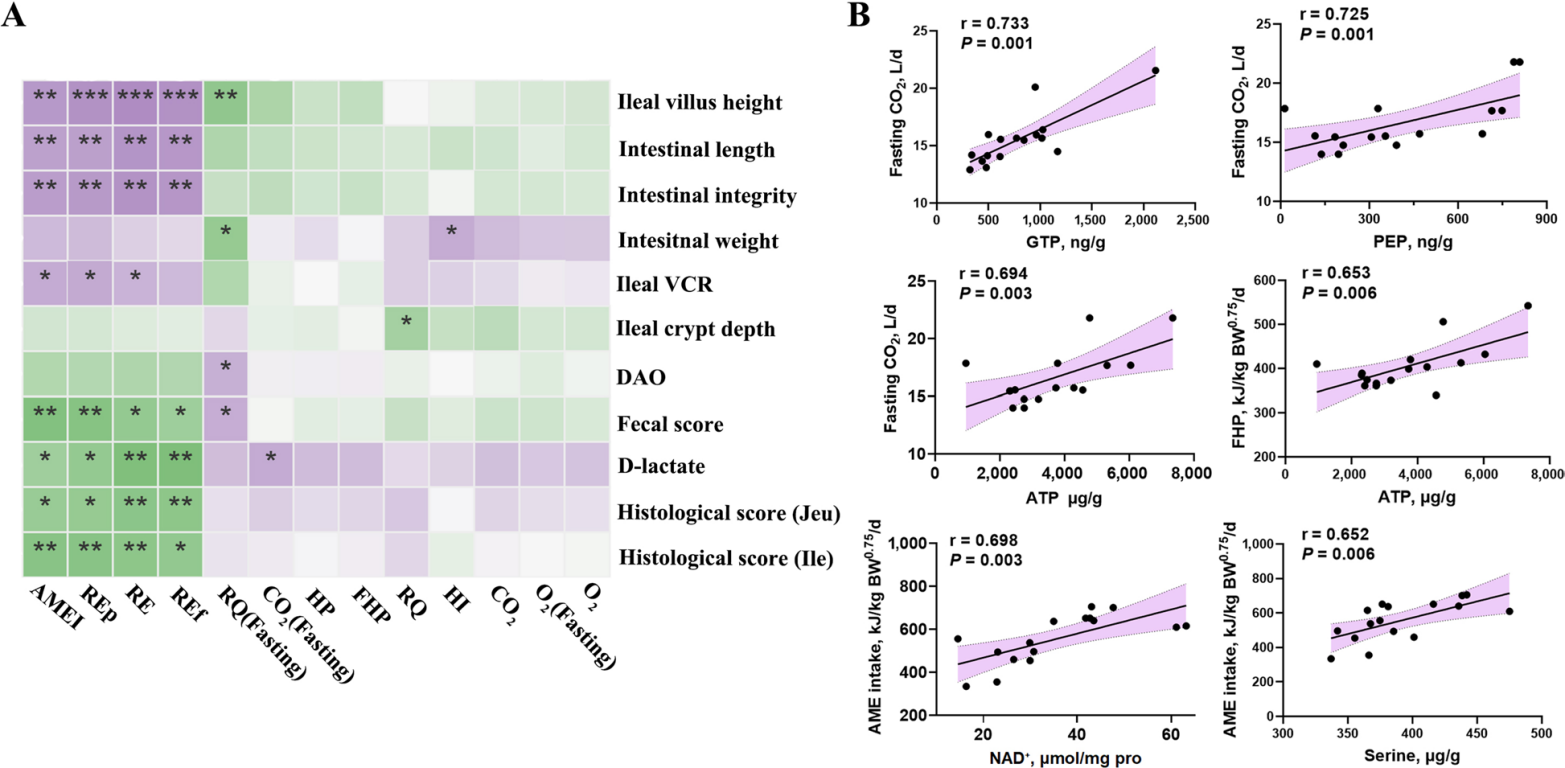
Correlation analysis of whole-body energy balance with inflammatory status and energy metabolites in ileal mucosa. **A** Pearson correlation between whole-body energy balance and inflammatory status. The purple squares represent positive relationships (*r* > 0), and green squares indicate negative relationships (*r* < 0). **B** Linear regression showing the top 6 relationships with relatively high correlation coefficient. The error bands are shown, with the purple area filling within these bands. ^*^P < 0.05; ^**^P < 0.01; ^***^P < 0.001

Pearson correlation analysis between whole-body energy balance and inflammation status was performed (Fig. 7A). The D-lactate content in serum showed a positive relationship with CO_2_ production in fasted birds (*P* < 0.05). RQ in fasted birds was positively (*P* < 0.05) correlated with serum diamine oxidase (DAO) content and the fecal score, while negatively correlated with the ileal villus height and intestinal weight (*P* < 0.05). In fed birds, RQ exhibited a negative association with ileal crypt depth (*P* < 0.05). As expected, HI increased in parallel with intestinal weight (*P* < 0.05). Moreover, AME intake, RE, RE as fat and RE as protein were inversely (*P* < 0.05) related to serum D-lactate content, jejunal and ileal histological scores, and average fecal score. In contrast, these parameters displayed positive (*P* < 0.05) relationships with total intestinal length, ileal villus height, ileal VCR and intestinal integrity score, although the relationship between ileal VCR and RE as fat was not significant (*P* > 0.05).

Moreover, the Pearson correlations between energy metabolites in ileal mucosa and whole-body energy homeostasis are shown in Fig. S1. The top six relationships ranked by* r* value were further identified by using linear regression, with error bands shown in purple (Fig. 7B). Fasting CO_2_ production showed positive correlations (*P* < 0.05) with GTP (*r* = 0.733), PEP (*r* = 0.725) and ATP (*r* = 0.694). Positive relationship was observed between FHP and ATP (*P* = 0.006, *r* = 0.653). Moreover, AME intake was positively associated (*P* < 0.05) with NAD^+^ (*r* = 0.698) and serine (*r* = 0.652) contents in ileal mucosa.

## Discussion

This study assumed that DSS induction impaired intestinal barrier function and caused a shortage of available energy, thereby adversely affecting the metabolism of whole body. Generally, inflammation may induce the infiltration of inflammatory factors and the recruitment and aggregation of immune cells, thereby increasing the HP of animals [17]. In our study, intestinal inflammation did not lead to a significantly higher body HP, possibly due to the numerically reduced feed intake and the shortened intestinal length induced by DSS. A reduction in intestinal weight could proportionally reduce HI (Fig. 7A) and the basal energy requirements [30]. The effect of feed intake on HP was also reported by Campos et al. [31], who observed decreases in AME intake and HP related to reduced feed intake following an LPS challenge in growing pigs, even though the presence of typical inflammatory symptoms. Moreover, because of the limited chamber size and instability of calorimetry data, some meaningful changes in HP may be covered up by individual differences. Specifically, the basal metabolic rate, reflected by FHP, slightly rose from 391.74 to 417.69 kJ/kg BW^0.75^/d following enteritis induction. FHP values fell within the range of 376.52 to 499.15 kJ/kg BW^0.75^/d reported by Liu et al. [32] and Sakomura et al. [33] in broiler chickens. Furthermore, FHP accounted for approximately 75% of the total HP (Fig. 3), equivalent to the documented values in hens [34].

Since HI was unaffected by enteritis induction, it can be speculated that intestinal energy metabolism of birds in fasted state is more sensitive to inflammatory processes than in fed state. The inflammatory status was significantly correlated with RQ and CO_2_ production of pullets in fasted state, but not in the fed state (Fig. 7A). The impact of inflammation on RQ may be masked by heat production during feeding. Generally, the RQ values reflect the primary substrate being metabolized: a value of 0.7 suggests lipid catabolism, 0.8 indicates protein catabolism and 1.0 indicates glucose metabolism. RQ value fluctuates around 1.0 during feeding and approaches 0.7 after fasting, because metabolism shifts from carbohydrate to fat oxidation [35]. Therefore, the RQ values of 0.80 and 0.74 in fasted birds potentially suggest that body fat and protein are mobilized to generate energy, especially after intestinal inflammation.

Distribution of ME intake may help to explain the direction and magnitude of energy flow in the body following intestinal damage. As expected, AME intake decreased following DSS induction mainly because of the numerically reduced appetite (−20.07%) and impaired utilization of dietary energy. The proportion of HP relative to AME intake increased from 60.14% to 90.61% following DSS induction (Fig. 3). Consistently, immune stimulation led to a 23% increase in energy allocation to the immune system [17]. In the CON group, energy retention in birds was allocated as 83.04% fat and 16.96% protein, comparable to values reported in the literature (85% fat and 15% protein) by Boekholt et al. [36]. However, the ratio of RE to AME intake was negative (−18.81%) under inflammatory conditions, suggesting that birds likely consumed body energy to respond to inflammation. The amount of fat deposition can be estimated by dividing RE as fat by its energy content of 38.9 kJ/g [37]. Hence, it could be speculated that fat deposition was negative in the INFL group, in parallel with the negative RE as fat (−144.64 kJ/kg BW^0.75^/d). Partly consistent with the current study, under low energy intake, energy was primarily deposited as protein in broilers at the expense of fat consumption [36]. Overall, enteritis induction compromised AME intake but slightly increased the basal metabolic rate in pullets, potentially resulting in the mobilization of body fat to meet energy demands.

Contrary to previous studies in piglets [7] and in broilers [38], enteritis induction reshaped adenine nucleotide pool and enhanced both ATP level and AEC in ileal mucosa, reflecting maximized ATP production in inflamed intestine. Differences in inflammation models and inflammatory phases may account for the observed variations. Consistent with the results observed in mice, the intestine showed increased ATP production after colitis induction to support energy demand [39]. However, DSS-induced reduction in intestinal length may compromise the ATP pool [40]. Beneficially, ATP can fuel actomyosin turnover and facilitate the renewal of tight junction proteins, thereby promoting the restoration of intestinal barrier function [41]. Besides, the elevation of high-energy phosphate molecules indicates substantial alterations of intestinal energy substrates and associated pathways.

The continuous turnover of NAD⁺/NADH is crucial for ATP production and energy homeostasis. Enteritis-induced decrease in NAD⁺ and NAD⁺/NADH ratio suggests that NADH is unable to be effectively oxidized back to NAD⁺, potentially due to the mitochondrial disfunction and the impaired ETC following intestinal injury [42]. Indeed, inflammation can trigger a unique metabolic state that rapidly depletes NAD⁺ by increasing glycolysis and stimulating DNA repair and synthesis [43]. Specifically, these processes are highly dependent on nucleotides like dTMP and dAMP, which were found to be enriched following enteritis induction in this study. Precious study also illustrated an impaired NAD^+^ metabolism during chronic infections and inflammation [44]. Given unbalanced NAD⁺/NADH ratio coincide with increased ATP content in ileum, enteritis induction impaired ETC in ileum, potentially switching energy production to alternative pathways.

Glycolysis is a major metabolic pathway that converts glucose into pyruvate and glucose is an essential fuel for the immune system [9]. As expected, glycolytic intermediates including F-1,6-BP, DHAP, 3-PG, 2-PG and PEP were increased to varying degrees in ileal mucosa, reflecting an enhanced glycolytic flux. This partially aligns with that inflammation promoted glycolysis [45], similar to the “Warburg” effect observed in tumor cells. Importantly, the enrichment of downstream glycolytic metabolites may compensate for ATP generation during inflammation (Fig. 5C), despite it is far less efficient than oxidative phosphorylation [8]. Moreover, the ATP-dependent conversion of fructose-6-phosphate (F-6-P) to F-1,6-BP, catalyzed by PFK, was restricted possibly in response to cell conditions and high demand for ATP[46]. The accumulation of 3-phenyllactate was observed in inflamed ileum (Fig. 4B), consistent with previous findings reported in LPS-challenged broiler chickens [47]. Furthermore, it could be considered as a metabolic marker of intestinal inflammation [48]. Moreover, PPP is an important branch pathway of glycolysis, and its substrates such as S7P and glucuronic acid can replenish metabolic pool of glycolysis in response to intestinal inflammation. Another plausible explanation for the reduction in PPP metabolites is the utilization of pentoses in nucleotide synthesis for tissue repair [49]. Therefore, the elevation of glycolytic substrates possibly suggests that glycolysis plays a role in energy production following intestinal inflammation.

TCA cycle is the central pathway for the oxidation of fats, carbohydrates and proteins. The accumulation of α-KG level attributed to the suppression of *α-KGDH* and the depletion of NAD^+^ potentially indicates a disruption of TCA cycle. Specifically, α-KGDH couples the conversion of α-KG to succinyl-coA, a process driven by NAD^+^ (Fig. 6B). Moreover, α-KG could be replenished by glutamine and glutamate in response to the metabolic changes during inflammation. Apart from *α-KGDH*, other NAD^+^ or FAD dependent dehydrogenases like *IDH3A* and *SDHB*, were also downregulated during inflammation, thereby suppressing the TCA cycle [50]. Similarly, reduction in TCA cycle enzymes was observed in mice challenged with DSS [51]. Previous study reported that dietary α-KG inclusion could ameliorate DSS-induced colitis by suppressing inflammation and reshaping microbial composition [52]. Our study reveals that enteritis induced intestinal metabolic remodeling to accumulate α-KG levels, which may help restore intestinal integrity.

During intestinal inflammation, alanine, serine and threonine may be converted to pyruvate for energy production via entering TCA cycle (Fig. 6B). Additionally, asparagine, glutamine and tyrosine are precursors of oxaloacetate, α-KG and acetyl-coA, respectively (Fig. 6B), fueling for the TCA cycle to produce energy. Amino acids such as phenylalanine and tyrosine can be transformed into 3-phenyllactate in an anoxic mucosal environment, and the enrichment of 3-phenyllactate after DSS induction, in turn, implies increased phenylalanine catabolism [48]. Collectively, almost all amino acids detected in this study were mobilized and participated in energy supply during intestinal inflammation.

Pearson correlation further confirms that ATP generation is accompanied by CO_2_ production in fasted state, and that the maintenance energy requirement (equal to FHP) is essentially an ATP requirement [14]. Indeed, intestinal inflammation remodels metabolic pathway to maximize ATP production in intestine, accompanied by increased CO_2_ and HP in other organs and throughout the body (Fig. 7B) [53]. Enteritis-induced reduction in energy intake may decrease available energy in the intestine, coupled with NAD⁺ depletion (Fig. 7B). Hence, respiratory data may serve as potential indicators to predict the intestinal energy status of pullets. Moreover, supplemental with energy metabolites like NAD-related substrates and several amino acids may restore intestinal health by alleviating intestinal energy deficiency. It is important to mention that the model, duration and intensity of inflammation may also impact energy metabolism. Moreover, a pair-fed trial is necessary to comprehensively assess the relationship between intestinal energy metabolism and whole-body energy balance, which requires further investigation. Other techniques should be combined, such as oxygen difference and radioisotope turnover method, to determine O_2_ consumption and complex metabolic processes in inflamed intestine.

## Conclusion

Intestinal inflammation induced by DSS caused disruption of energy metabolism in ileal mucosa, evidenced by depletion of amino acids, blockage of TCA cycle and enhanced glycolytic metabolites. Moreover, these modifications coincided with both enhanced ATP contents and an imbalanced NAD^+^/NADH ratio in ileal mucosa. Although HP was not significantly affected, inflammation compromised AME intake (−190.47 kJ/kg BW^0.75^) of pullets, leading to consumption of body energy reserves (−18.81% of AME intake) to maintain energy homeostasis. The inflammatory parameters were closely correlated with energy intake, energy partitioning, fasting RQ and CO_2_ production in pullets. Moreover, respiratory data may serve as potential indicators to predict intestinal energy status in layer pullets.

## Supplementary Information

Additional file 1: Fig. S1. Correlations between energy metabolites in ileal mucosa and whole-body energy homeostasis.

## Data Availability

The original contributions presented in the study are included in the article, further inquiries can be directed to the corresponding author.

## Abbreviations

ADP: Adenosine diphosphate
AEC: Adenylate energy charge
AME: Apparent metabolizable energy
AMP: Adenosine monophosphate
ATP: Adenosine triphosphate
ATP5A: ATP synthase F1 subunit alpha
ATP5B: ATP synthase F1 subunit beta
CON: Control
CS: Citrate synthase
dAMP: Deoxyadenosine monophosphate
DAO: Diamine oxidase
DHAP: Dihydroxyacetone phosphate
DM: Dry matter
DSS: Dextran sulfate sodium
dTMP: Deoxythymidine monophosphate
F-1,6-BP: Fructose-1,6-bisphosphate
F-6-P: Fructose-6-phosphate
FHP: Fasting heat production
GTP: Guanosine triphosphate
HI: Heat increment
HK: Hexokinase
HP: Heat production
IDH3A: Isocitrate dehydrogenase [NAD(+)] 3 catalytic subunit α
INFL: Inflammation
LGR5: Leucine-rich repeat-containing G-protein coupled receptor 5
MUC2: Mucin 2
NAD: Nicotinamide adenine dinucleotide
PEP: Phosphoenolpyruvate
PFK: Phosphofructokinase
PGK2: Phosphoglycerate kinase 2
PK: Pyruvate kinase
PPP: Pentose phosphate pathway
RE: Retained energy
S7P: Sedoheptulose-7-phosphate
SDHB: Succinate dehydrogenase B
SUCLA2: Succinyl-CoA ligase ADP-forming α subunit
TAN: Total adenine nucleotides
TJP1: Tight junction protein 1
VCR: Villus height to crypt depth ratio
α-KGDH: α-Ketoglutarate dehydrogenase complex
2-PGA: 2-Phosphoglycerate
3-PGA: 3-Phosphoglycerate
