# Supplementary material for: Intestinal inflammation disrupts energy metabolism in layer pullets: insights into energy partitioning and intestinal metabolomic profiling
Source: J Anim Sci Biotechnol. 2025 May 26;16:76. doi: 10.1186/s40104-025-01204-x (PMC12105190; doi:10.1186/s40104-025-01204-x)
Supplement: Supplementary file 1 — Additional file 1: Fig. S1. Correlations between energy metabolites in ileal mucosa and whole-body energy homeostasis. [file 40104_2025_1204_MOESM1_ESM.docx]

**
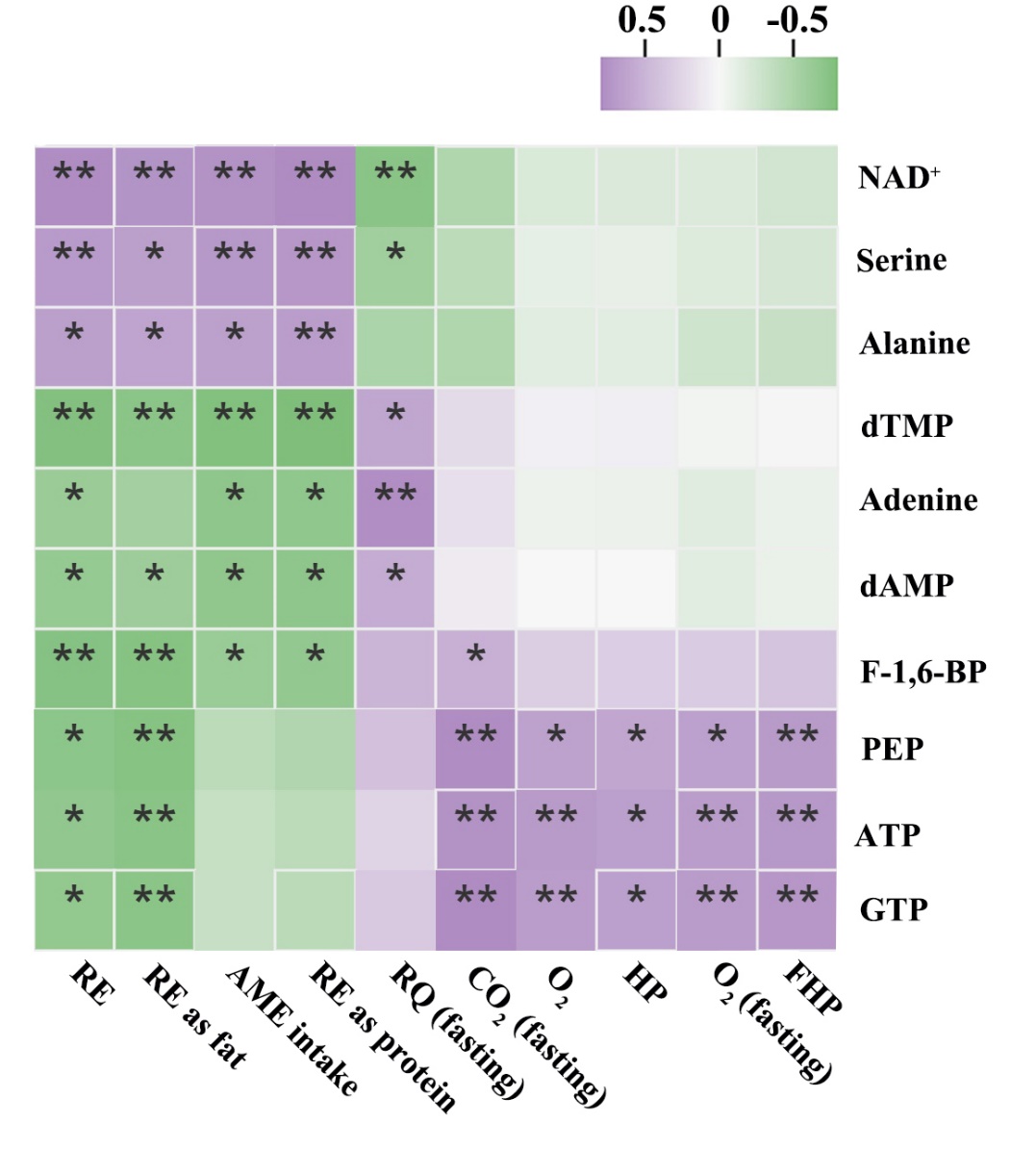
**

**Fig. S1** Correlations between energy metabolites in ileal mucosa and whole-body energy homeostasis. Heatmap displays the Pearson correlations filtered by criteria (*r* > 0.60 or < −0.60 and *P* < 0.05). The purple squares represent positive relationships (*r* > 0), and green squares indicate negative relationships (*r* < 0)
